# Go Girls!—Dance-Based Fitness to Increase Enjoyment of Exercise in Girls at Risk for PCOS

**DOI:** 10.3390/children6090099

**Published:** 2019-09-06

**Authors:** Anna K. King, Kara McGill-Meeks, Jennifer P. Beller, Christine M. Burt Solorzano

**Affiliations:** 1 Department of Pediatrics, Children’s Fitness Clinic, University of Virginia, Charlottesville, VA 22908, USA; 2 Augusta Health, Outpatient Diabetes and Nutrition Education Program, Waynesboro, VA 22939, USA; 3 Saratoga Hospital Medical Group, Endocrinology and Diabetes, Wilton, NY 12831, USA; 4 Center for Research in Reproduction, University of Virginia, Charlottesville, VA 22908, USA

**Keywords:** Zumba, dance, polycystic ovary syndrome, childhood obesity

## Abstract

Weight loss can reduce the hyperandrogenemia associated with polycystic ovary syndrome (PCOS) in peripubertal girls. Yet, adolescent girls have the lowest rates of physical activity and enjoyment of exercise. We created a dance-based support group (Go Girls!) to entice physical activity and improve enjoyment. Girls ages 7–21 over the 85th BMI percentile were recruited and attended once-weekly sessions for 3–6 months. We assessed changes in Physical Activity Enjoyment Scale (PACES), anthropometrics, laboratory data, and amounts of home exercise at 0, 3, and 6 months. Sixteen girls completed either 3 or 6 months. PACES scores were surprisingly high at baseline and remained high. Systolic blood pressure percentile decreased post-intervention. Although no group differences were observed, the majority of individual girls had decreased waist circumference, triglycerides, and metabolic syndrome severity score. Forty percent had decreased free testosterone levels. More girls enjoyed physical education class, got exercise outside of school, and made other lifestyle changes. This dance-based support group was enjoyed by girls and demonstrated health benefits. Continued efforts to engage girls in physical activity are necessary to protect girls from the consequences of obesity, including PCOS and metabolic syndrome. Dance exercise remains a promising tool to encourage physical activity in girls.

## 1. Introduction

Hyperandrogenic polycystic ovary syndrome (PCOS) affects 8–10% of all reproductive-aged women and is the leading single cause of female infertility, associated with serious metabolic problems, including obesity, type 2 diabetes mellitus/gestational diabetes, dyslipidemia, obstructive sleep apnea, hypertension, and preeclampsia [1]. National medical costs for PCOS and its co-morbidities were estimated to be over $4 billion annually in the U.S.A. in 2004 [1] and at least £237 million annually in the U.K. in 2018 [2], mostly related to increased rates of diabetes. Since excess androgen production during puberty may be a forerunner to adult PCOS, interventions to prevent PCOS aimed at adolescent girls are poised to have a substantial effect on this disorder.

PCOS prevalence is enhanced with obesity, rising to 65% in some populations of overweight women [3]. Peripubertal obesity in girls has been associated with hyperandrogenemia in most [4,5,6], although not all [7], studies. Reinehr et al., found that obese pre- and pubertal girls had total testosterone levels that were 4- and 1.75-fold elevated and sex hormone binding globulin that were 26% and 44% lower, respectively, compared to normal-weight girls. In these obese girls, weight loss improved testosterone levels towards control values [4]. In our studies, obese pre- to mid-pubertal girls had free testosterone levels that were 5-fold higher [5] and hyperandrogenemia occurred in ~65% of obese girls at every stage of puberty [6]. Currently, 27.8–47.9% of U.S. children and adolescents aged 6–19 are overweight or obese, with the highest rates observed in adolescents [8]. Healthy diet and exercise are the primary treatments for weight management and prevention/treatment of co-morbidities in children/adolescents, with the 2017 U.S. Preventative Services Task Force on Obesity prescribing 26 h of behavioral lifestyle modification intervention for any child with obesity [9]. Furthermore, weight loss due to lifestyle intervention with exercise training, nutrition education, and behavior therapy can be effective at reducing hyperandrogenemia in prepubertal girls and adolescents with PCOS [10,11], and physical activity alone can decrease testosterone in women with PCOS [12]. This is most likely via reducing insulin resistance and may thus act in a manner similar to other PCOS treatment strategies that reduce hyperinsulinemia, such as diazoxide, octreotide, D-chiro-inositol, metformin, or thiazolinediones [13,14]. 

Unfortunately, adolescent girls participate in physical activity at lower rates than children of any other demographic group [15,16]. Strategies to enhance enjoyment of physical activity have increased activity levels in low-active adolescent girls [17]. These studies assessed exercise enjoyment using the Physical Activity Enjoyment Scale (PACES) [17,18], a 16-question survey using a scale of 1 to 5. Girls with a low baseline enjoyment of exercise showed greater increases in vigorous activity compared to girls with high baseline enjoyment, suggesting that the low-enjoyment group may benefit most from interventions targeting barriers to physical activity. 

Intrinsic motivation for healthy habits like exercise are increased by feelings of competence, autonomy, and relatedness [19]. Exercise interventions including peer support [20] have improved outcomes for adolescents, especially when there is community involvement in the programs [15,16]. Similarly, group office visits have been demonstrated to improve health outcomes in adults with diabetes mellitus and coronary artery disease [21], diseases that also require lifestyle changes for effective treatment. 

To address the serious barriers identified in the University of Virginia (UVA) Pediatric clinics for families implementing exercise lifestyle changes—specifically, a paucity of safe spaces to exercise for minimal cost—and to increase delivery of the critical messages of how lifestyle affects overall and reproductive health, we utilized dance exercise to develop a free, fun, and engaging exercise/education support group for girls. Our hypothesis was that the Go Girls! program, intended to introduce girls to a fun, non-threatening environment of exercise with peer support, would improve scores on a physical activity enjoyment scale (PACES).

## 2. Materials and Methods

Go Girls! Fitness Support Group. Go Girls! is a dance-based fitness support group developed in collaboration with the UVA Children’s Fitness Clinic and the UVA Center for Research in Reproduction as a PCOS prevention and treatment program for girls ages 7–21 years and their female caregivers (e.g., mothers, grandmothers, aunts, neighbors). We targeted the Go Girls! program to (but did not limit it to) those who are at-risk for weight-related problems, in particular those with PCOS, diabetes mellitus, or insulin resistance. We held weekly sessions consisting of 45 min of dance-based exercise (primarily Zumba^®^ Fitness or Kukuwa^®^ African Dance) followed by 15–20 min of educational discussion about health-related topics, including complications of insulin resistance and PCOS. UVA undergraduate, medical, and nursing students, UVA pediatric resident physicians, UVA Medical School and Curry Education School faculty and staff, and community experts (e.g., female Virginia State Police officers) led discussions. 

Selected topics discussed by theme:

- Benefits of Exercise—protection against consequences of inactivity and obesity; positive effects on mood, attention, sleep, bone strength, cardiovascular and reproductive health
- How to Develop Exercise Habits—types of exercise, developing a routine, safety, staying motivated, DIY home exercises, strength training, pedometer apps, appropriate screen time
- Nutrition—healthy holiday eating, healthy beverages, healthy snack cooking demonstration/taste test, portion control, eating seasonal produce
- Preventative Medicine—bone health, PCOS
- Interpersonal relationships—random acts of kindness; how to be a good friend
- Mental health—self-esteem activities, positive body thoughts, positive self-talk, media reality check
- Social well-being—personal safety, self-defense, internet safety
- Intellectual development—higher education, leadership, careers in science fields

We worked with participants on “SMART” (Specific, Measurable, Achievable, Relevant, Time-Bound) goal setting and attainment for lifestyle modification. This was carried out via one-on-one assistance with constructing goals, fitness challenges with prizes, focus groups discussing how to overcome barriers to lifestyle changes, and poster-sized class charts with group goals to increase intrinsic motivation. We engaged girls in the groups by encouraging them to choose songs and to develop choreography for the sessions, emphasizing enjoyment of activity, exploring social media opportunities for class discussion/encouragement, and developing a network of undergraduate volunteers to serve as mentors. We partnered with a local health-conscious restaurant (Roots Natural Kitchen) to provide cooking demonstrations/taste tests for participants.

Assessment of success. Any girl participating in the Go Girls! program with a body mass index-for-age percentile (BMI%) ≥ 85 was approached to join an optional efficacy study. We evaluated the efficacy of the Go Girls! Fitness and Education Support group in the following ways: (1) we scored enjoyment of exercise via the PACES questionnaire [17,18], (2) we evaluated anthropometrics with optional fasting blood work at baseline, 3 months, and 6 months, and (3) we assessed attitudes towards exercise and quantity of home exercise. Attendance challenges (with prizes) with focus group discussions every 2 months helped to motivate, as well as track, individual and program progress. PACES is scored on a scale of 1 to 5, with reports demonstrating baseline enjoyment of 2.75 (SD = 0.46) in 29 girls with low enjoyment of activity [17]. We assessed changes in blood pressure, body mass index percentile-for-age, and waist circumference in all subjects, as well as fasting serum-free testosterone (calculated from total testosterone and sex hormone binding globulin), dehydroepiandrosterone sulfate, insulin, glucose, HbA1c, and lipids in subjects who agreed to optional fasting blood sampling. Amounts of average daily moderate-vigorous physical activity (e.g., walking, jogging, playing active games, biking) were assessed by a survey of the number of minutes doing activity outside of gym class per week (0–30 min, 31–60 min, 61–90 min, 91–150 min, >150 min). We obtained qualitative responses on their readiness for change, their attitudes about physical education class, whether they thought their parents’ level of activity affected their own physical activity, what “physical activity” means to them, and overall comments about the program. 

Analysis. *Sample size*. Since we targeted our program towards adolescent girls with excess weight and weight-related medical problems, we assumed that they would also have low enjoyment of exercise scores at baseline. Based on a two-tail power calculation, with a test value of 2.75 and SD 0.46, we needed 20 subjects to detect a 15% difference in enjoyment scale score with a power of >80% (*p* > 0.05). *Calculations*. Percentiles for BMI and blood pressure were calculated using an online calculator based on the U.S. Centers for Disease Control and Prevention (CDC) growth charts (http://www.quesgen.com/BMIPedsCalc.php). Homeostasis model assessment of insulin resistance (HOMA-IR) was calculated using the formula: fasting insulin (µU/L) * fasting glucose (mmol/L)/22.5 [22]. Metabolic Syndrome Severity (MetS) z-scores (https://metscalc.org/metscalc/) were derived from confirmatory factor analysis, examining how the components of metabolic syndrome (BMI, systolic blood pressure, fasting triglycerides, HDL cholesterol, and fasting blood glucose) are correlated to one another and vary by sex and race/ethnicity (Hispanic, non-Hispanic black, non-Hispanic white) [23]. The score has a normal distribution with a mean of 0 and standard deviation of 1, was devised based on nationally representative National Health and Nutrition Examination Survey (NHANES) data from the CDC, and correlates strongly with long-term risk of cardiovascular disease and type 2 diabetes mellitus [24,25]. *Statistics*. In our primary analysis, we assessed for differences in PACES score pre- versus post-intervention for girls completing either 3 or 6 months using the Wilcoxon Signed-Rank nonparametric analog to the paired *t*-test (SAS 9.2) with the latest data obtained for each girl. Secondary analyses assessed for pre- versus post-intervention differences in anthropometric and laboratory values using the same statistical strategy. We also assessed for group differences between the enrolled girls and the girls at baseline who eventually completed the study using Student’s *t*-tests. We analyzed for differences in frequency of metabolic syndrome pre- versus post-intervention using Fisher’s Exact test. 

Human Subjects. All subjects gave their informed consent for inclusion before they participated in the study. The study was conducted in accordance with the Declaration of Helsinki, approved by the UVA Health Sciences Research Institutional Review Board (IRB-HSR16132), and listed on ClinicalTrials.gov (NCT02117063). Subjects who agreed to laboratory analysis were given $25 per blood draw. Participation in the Go Girls! program was free to all girls and caregivers.

## 3. Results

Program. From 2013 to 2018, Go Girls! fitness support programs were developed near the UVA Health System and surrounding regions. Sites included the UVA Children’s Hospital (287 girls), UVA Orange Pediatrics outreach clinic 30 miles northeast (129 girls), Augusta Health (a non-UVA-affiliated medical center) at the Waynesboro YMCA 40 miles west (19 girls, 2014–2016 only), and UVA Zion Crossroads Specialty clinic 18 miles east (16 girls, 2017–2018). In an attempt to extend the program to more remote areas, we piloted the use of UVA telemedicine, but participants strongly preferred in-person group leaders.

**Figure 1:**
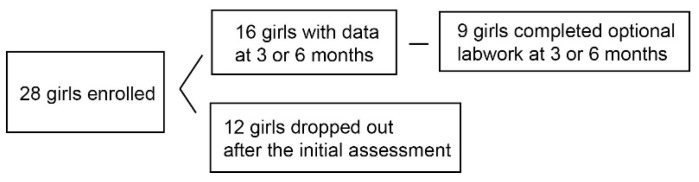
Subjects recruited and retained.

Study participants. Twenty-eight girls were enrolled in the efficacy study (age 13.1 y ± 2.7 [mean ± SD], range 8–17) (Figure 1, Table S1), of which 29% were non-Hispanic black and 11% were Hispanic. Fourteen of these girls agreed to optional fasting serum laboratory studies. In addition to obesity, diagnoses in the girls included PCOS, hyperlipidemia, pre-diabetes, type 1 diabetes mellitus, insulin resistance, hypertension, irritable bowel syndrome, ulcerative colitis, asthma, anxiety, bipolar disorder, attention deficit disorder, and post-traumatic stress disorder. Seven had a first degree relative with type 2 diabetes mellitus. Parents also had histories of coronary artery disease, kidney stones, depression, hypertension, type 1 diabetes mellitus, and cancer. There was a high rate of drop-out from the study: 16 girls (9 with serum samples) and 11 girls (8 with serum samples) completed 3 and 6 months, respectively, giving 43% and 61% drop-out rates at 3 and 6 months. Post-intervention data were analyzed using the latest follow-up assessments available for all girls who completed either 3 or 6 months of the program (*n* = 16) (Table 1; Figure 1; Table S1). 

PACES survey. On average, the girls recruited had a high enjoyment of exercise PACES score at baseline, which varied between individuals (mean 4.03 ± 1.01, range 1.19–5.00) (Table 1; Table S1). Sixty-four percent of girls had scores >4.00 points. PACES scores increased slightly, but not statistically, in girls who had follow-up data (pre: 3.88 ± 1.25 vs. post: 4.11 ± 1.06, *p* > 0.1). The girl with the lowest enjoyment score at baseline completed 6 months with the biggest increase in score (1.19 to 2.69).

**Figure 2:**
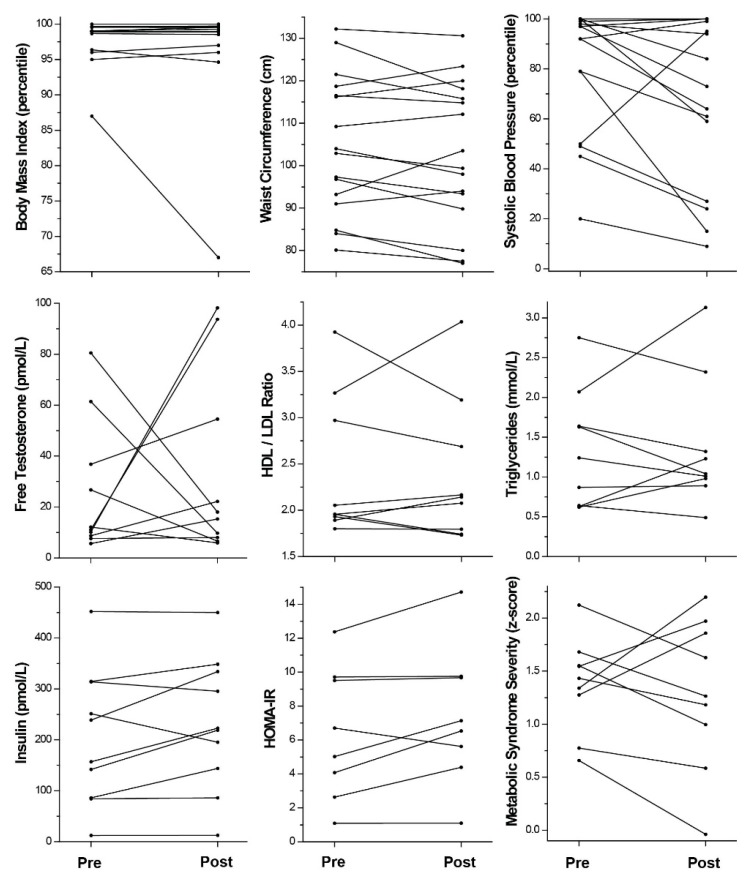
Individual results.

Anthropometrics. All the girls were overweight on study entry, ranging from BMI% 87 to 100 (BMI% 98.2 ± 2.6; BMI 34.3 ± 7.2 kg/m^2^, range 21.5–52.7; BMI z-score 2.26 ± 0.40; 1.13 to 2.88). The average baseline BMI% for girls completing versus enrolled in the study was similar (Table 1; Table S1). For girls completing the study, weight remained the same (delta: +1.0 kg ± 4.3, range −8.4 to +9.5, *p* > 0.1). BMI, BMI%, or z-score was not different post-program (*p* > 0.1 for all) (delta: 0.513 ± 1.8 kg/m^2^, range −3.2 to +4.7; −1.2% ± 5.1, range −20 to +1; 0.00 SD ± 0.22, range −0.70 to +0.304). Although the majority of girls gained weight (up to 9.5 kg), some individuals demonstrated successful weight loss: 5 girls lost weight and absolute BMI at 3 months, 4 lost at 6 months (Figure 2). One young woman normalized her BMI% by 3 months and decreased further from 87% at baseline to 67% at 6 months.

Systolic blood pressure was abnormal (>90%-ile for sex, age, and height) in 44% of the girls enrolled (120.2 mmHg ± 13.4, range 102–150; 76.8% ± 23.3, range 20–100; 4.13 z-score ± 15.6, range −0.84 to 3.74). The average blood pressure absolute value and percentile were not significantly different for girls completing versus enrolled in the study (Table 1; Table S1). Notably, after the intervention, systolic blood pressure percentiles were significantly lower (*p* = 0.04) (delta: −12.6% ± 24.1, range −64 to +45) in girls completing the study. Absolute systolic blood pressure also appeared to decrease but did not reach statistical significance (delta: −4.8 mmHg ± 15.9, range −19 to +13, *p* > 0.1). Sixty-seven percent lowered their systolic blood pressure percentiles (Figure 2) by at least 4%; four girls with previously abnormal blood pressures (>90%-ile) achieved values in the normal range.

Waist circumference was > 90th%-ile for age, race, and ethnicity [26] in 86% of girls recruited (105.9 cm ± 17.0, range 77.5–142.0 cm) (Table 1; Table S1). The average waist circumference in girls that completed the study was not significantly different from that of the girls enrolled. In the girls that completed the study, waist circumference did not change overall (delta: −1.8 cm ± 5.5, range −10.9 to +10.3, *p* > 0.1). Although the majority of girls (69%) had a decreased waist size, ranging from 1.6 to 10.9 cm lost (Figure 2), several girls increased their waist circumference, including one who gained 10.3 cm. Of those losing waist size, one subject normalized her waist circumference from 84.8 to 77 cm over 6 months and the girl with the largest benefit to waist size decreased from 129 cm to 118.1 cm over 3 months.

Laboratory assessments. Forty-seven percent of girls enrolled had elevated free testosterone levels (>17.4 pmol/L [>5 pg/mL], based on assay reference for adult women, since no normative values by age are available for children) (Table 1; Table S1). Free testosterone was not different after the exercise intervention in girls with follow-up data (pre: 26.1 pmol/L ± 26.0, range 5.6–80.5; post: 33.2 pmol/L ± 36.0, range 5.9–98.2). Although increases in free testosterone were observed in the majority of girls with changes ranging from 9.7 to 88.1 pmol/L, forty percent of girls completing the program lowered their free testosterone levels, ranging from −62.5 to −6.2 pmol/L (Figure 2). Two girls nearly normalized their free testosterone levels from 61.4 to 9.7 pmol/L and from 80.5 to 18.0 pmol/L. DHEA-S levels were not different in girls recruited than those who completed the program and didn’t change with the intervention (Table 1, *p* > 0.1).

Fasting insulin levels were elevated (>138.9 pmol/L [>20 uU/mL]) in 73% of the girls enrolled (199.0 pmol/L ± 110.6, range 40.3–452.1). Girls completing the program had similar baseline fasting insulin levels, which did not change significantly after the intervention (pre: 208.0 pmol/L ± 129.3, range 40.3–452.1; post: 233.3 pmol/L ± 126.4, range 38.2–450.0, *p* > 0.1) (Table 1; Table S1). Only a small number of girls (20%) had improvement in fasting insulin after the program (range of delta: −56.2 to −18.7 pmol/L) (Figure 2). Fasting glucose was normal in all girls, except one (5.67 mmol/L [normal < 5.55 mmol/L, 100 mg/dL]) who did not have follow-up data. Hemoglobin A1c was abnormal (≥5.7%) in 5 of 14 girls enrolled (2 had follow-up data), consistent with the diagnosis of pre-diabetes in youth and adults [27]. One normalized her A1c by 6 months (5.7 to 5.2%). Calculating HOMA-IR, 73% of enrolled girls had insulin resistance [≥3.4, [28]] with a mean of 6.1 ± 3.2 (range 1.1–12.4). Values were similar in girls completing follow-up (pre: 5.9 ± 3.9, range 1.1–12.4, *p* > 0.1) and did not change during the study (post: 6.8 ± 4.2, range 1.1–14.7, *p* = 0.1).

Fasting LDL/HDL ratios were elevated (>2.8) in 29% of girls enrolled (2.3 ± 0.7, range 1.4–3.9) (Table 1; Table S1). Overall, there was no change in LDL/HDL with the intervention. Some (33%) improved their ratios (range of delta: −0.7 to −0.2). Fasting triglycerides were also elevated (≥1.243 mmol/L [≥110 mg/dL]) in 43% of the girls enrolled (1.21 mmol/L ± 0.65, range 0.62–2.75), were not different in girls who completed (pre: 1.34 mmol/L ± 0.74, range 0.62–2.75, *p* > 0.1), and remained the same after intervention (post: 1.38 mmol/L ± 0.82, range 0.49–3.13, *p* > 0.1). Fifty-six percent improved their levels by at least 0.1 mmol/L during the program (range of delta: −0.59 to −0.15) (Figure 2)

Metabolic syndrome severity. Of the 14 enrolled girls, 11 (79%) had abnormal MetS z-scores (>1), with 1 girl having a score >2 (1.374 ± 0.463, range 0.657–2.123) (Table 1; Table S1). A score of 1 reflects the 84.1%-ile of U.S. children in the NHANES dataset, while a score of 2 reflects the 97.7%-ile [23]. MetS z-scores pre-intervention in girls with follow-up data were nearly identical to those enrolled (pre: 1.375 ± 0.447, range 0.657–2.123, *p* > 0.1). Although MetS z-score appeared slightly lower after study participation (post: 1.294 ± 0.715, range −0.039 to + 2.197), this did not reach statistical significance (*p* > 0.1). Scores decreased by at last 0.1 SD in 67% of girls (delta range −0.696 to −0.189) during the program, with one girl successfully normalizing her score (1.551 to 0.995) and one girl improving from >2 (2.123 to 1.628) (Figure 2). Still, another girl notably worsened to >2 (1.339 to 2.197).

Using traditional criteria, 50% of girls enrolled and 66% of girls completing the study met the definition of pediatric metabolic syndrome at baseline [29], with high rates of abdominal obesity, hypertension, and dyslipidemia (Table 2). After the program, the prevalence of metabolic syndrome seemed to decrease to only 22%, which did not quite reach statistical significance (*p* = 0.07).

Attitudes towards exercise/amount of exercise at home. At baseline, comments surrounding physical activity reflected generally positive attitudes, although 4 girls reported negative associations. All girls but one were “somewhat ready” or “completely ready” to get more exercise. Yet, one girl successfully completed 6 months of the program, despite persistently stating that she wasn’t ready for exercise. The majority enjoyed physical education class at school, specifically enjoying games and moving with friends. Although only 4 claimed to do any exercise outside of school, all the girls could name at least one type of exercise that they enjoyed. Negative comments included disliking fitness testing, time barriers, convenience barriers, and working out alone. By 3 and 6 months, almost all said they enjoyed physical education class and were exercising outside of school. Girls thought the program was very fun, especially the popular dance music. They reported making lifestyle changes in addition to exercise (e.g., drinking fewer sugary beverages, less screen time, watching portion sizes) and commented that the program helped them “feel better about themselves”. Family members described observing positive changes in the girls (e.g., more motivated, more organized, showing initiative at school) as a result of being part of Go Girls!. Two girls joined formalized sports teams after participation in the program. 

Barriers to participation. Barriers to participation reported were time constraints, distance, and personal conflicts, including academic time pressures and job opportunities as reasons for discontinuation. Transportation was difficult and time-consuming for several girls: some rode 1 ½ h each way, while others walked themselves along rural roads due to a lack of public or private transportation. Adults transporting girls became unavailable (e.g., parents divorced, after-school providers changed, family members became ill, and parents began working at night). Severe co-morbid physical or mental health problems also affected the ability to participate. One girl missed sessions after requiring hospitalization for ulcerative colitis. A family of three girls discontinued the program to better care for one girl’s severe anxiety after experiencing sexual abuse. Misconceptions about health also became a barrier: one girl’s mother forbade participation due to concerns for exercise-induced hypoglycemia when the girl developed pre-diabetes.

## 4. Discussion

This dance-based program was designed to entice girls into physical activity who are at-risk for health consequences of obesity, particularly PCOS, and who have low enjoyment of exercise. The girls recruited were the targeted population, as the majority was already experiencing PCOS and/or Metabolic Syndrome, complications of obesity. However, they had a surprisingly high baseline PACES score (4.03 ± 1.01) compared to previous studies in adolescent girls (2.75 ± 0.46) (17). After participation in this program, girls did report more enjoyment of physical education class and more exercise outside of school, as well as making other lifestyle modifications. However, no overall differences were observed in the PACES Enjoyment of Exercise questionnaire, our primary end-point. This lack of effect was likely due to a ceiling effect from the high baseline enjoyment scores. Girls who already enjoyed exercise may have self-selected to participate in our program, which is supported by how many girls stated that they liked PE class at baseline. Yet, girls reported very little exercise outside of school, possibly reflecting a lack of opportunities. Alternatively, baseline PACES values may have been inflated due to girls enjoying the first class and/or the novelty of the exercise, and may not have accurately represented their attitudes prior to participation. Girls may have overestimated enjoyment values to please study personnel who led the dance and support group activities. Lastly, although the questionnaire has been validated for use in adolescent girls [18], our sample included girls that were slightly younger, which may have affected their ability to understand all the questions, especially the negative ones. 

Unfortunately, we did not observe any improvements in mean androgen levels as a result of the program, although 40% of girls did experience a decrease in free testosterone levels. Two girls experienced increases in free testosterone (Figure 2), which may have been related to worsening obesity. Both had weight gain (2.45 and 6.58 kg) and increases in waist circumference (+2.9 and +3.8 cm), although not the largest individual increases in these obesity parameters. Two girls had notably decreased free testosterone, potentially related to decreased adiposity. Although one gained (+4.5 kg) and the other lost weight (−1.2 kg), both girls lost abdominal circumference (−1.7 and −5.4 cm, respectively), suggestive of improved visceral fat. This suggests a possible variable effect of exercise on body composition in individuals. The girl with improved free testosterone despite weight gain (and worsening HOMA-IR) had increases observed in her sex hormone binding globulin level. To observe greater improvement in hyperandrogenemia in girls with or at-risk for PCOS, the lifestyle modifications required may be more stringent than those needed to improve other measures of metabolic health, since we observed other health benefits from the program. 

We uncovered a concerning prevalence of hypertension in our young subjects at baseline, with 44% of girls having hypertension-based elevated age, sex, and height-adjusted percentile. The group mean absolute systolic blood pressure was also mildly elevated, even by adult standards. Notably, mean systolic blood pressure percentile—the only statistically significant finding—decreased as a group by the end of participation. Likewise, the majority of girls lowered their systolic blood pressure percentiles (even within the normal range) and several girls with hypertension at baseline normalized their blood pressures. These are important findings suggesting that a peer-supported exercise group may have particular benefits to cardiovascular health in youth. 

HOMA-IR was quite elevated in the girls in this study, indicating insulin resistance. This was not surprising, as we targeted the program to girls with weight-related co-morbidities, including PCOS, diabetes mellitus, and insulin resistance. Many of the girls were referred specifically by their primary care providers or by the obesity clinic due to concerns about risk for type 2 diabetes. Therefore, there was likely a referral bias towards more insulin-resistant girls. Our HOMA-IR results were similar to others studying obese children with insulin resistance [22,30,31]. Unfortunately, our data suggested that a program offering weekly exercise may be inadequate to prevent worsening of HOMA-IR in the majority of such insulin-resistant girls. 

Although there were no mean differences in anthropometrics or other lab indices, individual girls saw meaningful improvements in these parameters. Several girls lost weight, including one who normalized her BMI%, lowered her systolic blood pressure, and normalized her waist circumference. Unfortunately, we are not able to assess for improvements in this girl’s laboratory data for other metabolic risk parameters, since she opted not to give blood samples. The majority of girls who stayed in the program for at least 3 months saw improvements in systolic blood pressure, waist circumference, fasting triglycerides, and MetS z-score, all important markers of metabolic risk [29,32,33], including several girls who normalized or dramatically improved their values. Waist circumference can identify girls with high trunk fat mass by dual-energy X-ray absorptiometry [32] and, in women, it is a better predictor of coronary heart disease risk than BMI [34]. The MetS z-score was a useful tool to simultaneously evaluate changes in several physical and biochemical parameters to assess for metabolic risk improvement [23], which corresponded to the number of girls meeting criteria for metabolic syndrome pre- and post-intervention using more conventional criteria [29]. Although the prevalence of metabolic syndrome as a group did not quite reach statistical significance (*p* = 0.07), the improvements in MetS z-score seen in individual girls after program participation suggest that those individuals achieved decreased long-term risks of cardiovascular disease and type 2 diabetes mellitus [24,25]. 

Several important benefits of the program were not measurable by metrics. Dance interventions have been shown to have positive effects on body-related perceptions, self-trust, self-esteem, self-expression, and perceptions of dance abilities in children and adolescents [35]. With continued program participation, girls who initially avoided eye contact began smiling and routinely offering wellness ideas during discussion sessions; one such girl volunteered to make t-shirt logos for the group. Minority teens from a rural community who walked themselves to sessions volunteered to choreograph dances for the group. Mothers were appreciative of the safe space and positive messaging for their daughters while also commenting on improvements in their own waistlines. Girls progressed from sitting out due to deconditioning and embarrassment to dancing the whole time. Girls described enjoying school dances for the first time. Two girls joined organized sports teams (i.e., field hockey, soccer) after the program. Participants decided to represent the group by dancing down the streets in their town parade. Program staff noted more trusting relationships with their patients, the discovery of untapped talent and energies of colleague volunteers (as speakers, participants, and dance leaders), and the development of new bridges to outlying community organizations (including competing health systems, news stations, police department, farmers’ market organizers, and restaurants).

A major weakness of our study is the lack of a control group to evaluate changes in metabolic measures without the program; however, lifestyle changes for weight management are difficult to implement without structured support. Although not all variables improved after the intervention, it is possible that the exercise program prevented or ameliorated worsening in these parameters. Likewise, a control group would have better elucidated whether the PACES data was oddly high at baseline and described better the natural history of our primary end point in a longitudinal control cohort. Some variables may have improved simply by being observed, including blood pressure. The inclusion of an appropriate attention control group would have alleviated this concern, but resources available did not allow for careful ethical implementation of such a group. The design of this study would have also been improved if we had used validated scales for children to monitor food intake and exercise outside of the program. We also experienced low recruitment and retention numbers, which led to decreased power. Low recruitment occurred because we allowed program participation without study enrollment so that Go Girls! would be accessible to any interested girl. Recruitment of only overweight girls singled these girls out from lean peers and may have decreased their willingness to enroll. Parents of support group participants interested in the study were often unavailable to meet with study personnel for research consent. We anticipated a relatively high rate of drop-out from the program and the study, as has been previously observed in other weight loss interventions. Based on a similar community-based weight management program (Loozit^®^), which had a 14% attrition rate for its 2-month intervention [36], we had calculated a 42% attrition rate for a 6-month program. However, the Loozit^®^ program was implemented at an urban site, which may have improved access to program facilities. Despite offering the Go Girls! program in 3 off-site rural locations (up to 45 miles away from our medical center), girls still needed to travel considerable distances to reach the nearest group. This parallels the experiences of our patient population at UVA, who often travel up to several hours to attend clinic appointments. Additionally, extrapolating rates of attrition from the 2-month Loozit^®^ study may not have been appropriate for a program of longer duration. Yet, despite the drop-out, baseline anthropometric and biochemical values for girls completing the study were similar to those initially enrolled (Table 1).

The Go Girls! program also highlighted other unanticipated problems, which might be encountered by similar interventions. Around the medical center, we were one of several programs available to youth. We noticed that girls living near UVA were less invested in the program, possibly reflecting these additional opportunities available to them. In contrast, girls from outlying rural communities were very excited by this opportunity and attended more frequently, despite living further away. We piloted the use of telemedicine to better reach girls in rural settings, but this mode of instruction was not acceptable to girls or ancillary staff, who preferred in-person instructors. Therefore, we established auxiliary program sites in rural settings. Another problem was that dedicated exercise facilities were busiest during hours convenient for working families, which led to competition for class space and instructor time. We found that non-designated spaces near the clinic setting (e.g., conference rooms) were easier to access for these programs. Although barriers to lifestyle changes (or study participation) were not entirely unanticipated, we were dismayed and humbled that some were related to serious adverse childhood events (i.e., sexual abuse).

Despite these shortcomings, our experiences are congruent with other lifestyle interventions for children. Regarding subject recruitment, the Loozit^®^ study reported difficulty recruiting subjects, with only a 32% success rate per inquiry [37]. Despite improving girls’ physical activity levels, the Bristol Girls Dance Project found retention difficult as attendance steadily declined [38,39]. Still, encouraging dance as exercise is likely motivating and beneficial for girls. The Active Smarter Kids study demonstrated that girls prefer dance, gymnastics, exercising to music, and jumping rope to other physical activities [40]. Furthermore, a study of children in Ireland found that frequent dance club attendance was protective against obesity [41]. 

When determining the efficacy of intervention studies, reduction of body weight may not be the only parameter of value. Although there was no change in adiposity over 4 weeks, youth in the Dance for Health program achieved appropriate target heart rates while enjoying the dance exercise [42]. Moreover, physical activity intensity in girls may be more closely associated with cardiorespiratory fitness than with reductions in body fatness [40,43]. Childhood obesity experts remind us that “failure to reduce BMI should not be equated with failing to adopt healthier behaviors” that prevent metabolic and cardiovascular disease [44]. Indeed, one meta-analysis suggests that adding exercise to dietary advice interventions for children leads to greater improvements in metabolic parameters (i.e., HDL, fasting glucose, and fasting insulin) over 6 months [45]. Similarly to the increase in self-confidence observed in girls in our program, dance movement therapy improves mental well-being and total functioning for children and adults undergoing cancer therapy via creating an environment of shared positive experiences [46,47]. Indeed, several parents commented that our program created a much needed safe and positive environment to work on health and wellness goals.

After observing the time and transportation barriers to evening activities, we piloted similar dance-based education outreach programs (Go Girls and Guys!) to girls and boys enrolled in after-school and summer child care programs. We found that the Go Girls and Guys! programs consistently reached more children, as they brought fun exercise and health education to large groups of children already assembled while parents were still working. Additionally, the programs made use of existing space opportunities (i.e., school gyms), partnered with collaborative programs’ existing staff to supervise and model participation, developed more community partners, and also extended positive health messages to collaborative staff. Programs typically had 20–50 children per session. After such programs, 97% of youth could answer how lifestyle affects heath, 77% reported getting more daily exercise, 49% started a new physical activity, 43% made lifestyle changes, and 24% gained knowledge. Staff members were pleased that the topics reinforced health curricula and reported that children newly identified exercise in many different forms, including dancing. Therefore, the model of the Go Girls! program may be more effective if employed within environments already caring for children, as this would give more children opportunities to attend, improve long-term participation, and secure access to facilities for youth. Although adolescents typically do not attend such programs, encouraging pre-teen youth to consider dance for exercise may prevent the dip in enjoyment of exercise observed in adolescent girls.

## 5. Conclusions

The Go Girls! dance-based fitness support group provided fun physical activity to girls for free and demonstrated several important benefits. The program encouraged girls to achieve more physical activity outside of school, including involvement in organized sports. The primary health benefits observed were improved systolic blood pressure and a trend toward decreased prevalence of metabolic syndrome. Some individual girls reaped considerable benefits to body weight/adiposity, androgen levels, and metabolic syndrome severity score, with some girls achieving normalization of their metabolic risk. Wellness discussions inspired girls to make additional lifestyle modifications. The supportive atmosphere provided a positive environment where girls demonstrated increased self-confidence during physical movement and social engagement. Girls enjoyed the dance exercise program but had difficulty getting to sessions. Activities that are coordinated with other after-school programs may have more success in reaching more girls. Dance exercise remains a promising tool to encourage physical activity in girls. Continued efforts to engage girls in physical activity and promote healthy living are necessary to protect girls from the adverse consequences of obesity, including PCOS and metabolic syndrome.

This study also highlighted the idea that BMI is not always the most important outcome variable. Overall changes to health habits are critically important—especially participation in exercise that might continue beyond any specific program—since benefits (including in PCOS) can often be achieved before notable changes in obesity. 

## Figures and Tables

**Table 1:** Baseline and post-program subject data.

	Enrolled	Pre	Post
N	28 (14)	16 (9)	
Age (y)	13.1 ± 2.7	12.6 ± 3.0	
Race/Ethnicity (%)			
White	60	74	
Black	29	25	
Hispanic	11	1	
PACES (max 5)	4.03 ± 1.01	3.88 ± 1.25	4.12 ± 1.07
Body Mass Index (kg/m^2^)	33.6 ± 7.3	32.4 ± 6.9	32.9 ± 7.1
Body Mass Index (%-ile)	98.2 ± 2.6	97.8 ± 3.2	96.6 ± 8.0
Systolic Blood Pressure (mmHg)	120.2 ± 13.4	122.6 ± 15.9	117.8 ± 19.4
Systolic Blood Pressure (%-ile)	76.8 ± 23.3	80.3 ± 26.0	66.9 ± 33.5 *
Waist circumference (cm)	105.9 ± 17.0	104.8 ± 16.3	103.0 ± 17.0
Free Testosterone (pmol/L)	23.1 ± 21.9	26.1 ± 26.0	33.2 ± 36.0
DHEA-S (µmol/L)	3.99 ± 3.38	2.78 ± 1.98	2.80 ± 2.34
HDL (mmol/L)	1.05 ± 0.17	1.02 ± 0.14	1.01 ± 0.19
LDL (mmol/L)	2.29 ± 0.58	2.42 ± 0.68	2.36 ± 0.65
Triglycerides (mmol/L)	1.21 ± 0.65	1.34 ± 0.74	1.38 ± 0.82
HbA1c (%)	5.49 ± 0.30	5.38 ± 0.28	5.29 ± 0.35
Insulin (pmol/L)	199.0 ± 110.6	208.0 ± 129.3	233.3 ± 126.4
Glucose (mmol/L)	4.77 ± 0.45	4.55 ± 0.30	4.69 ± 0.35
HOMA-IR	6.09 ± 3.24	5.95 ± 3.88	6.80 ± 4.18
Metabolic Syndrome Severity (z)	1.374 ± 0.463	1.375 ± 0.448	1.294 ± 0.715

“N” is represented as total subjects (subjects with lab values). Data are mean ± SD, except race/ethnicity (%). * *p* < 0.05. SI to U.S. units: free testosterone pg/mL = pmol/L * 0.2882; DHEA-S µg/dL = µmol/L * 36.9; HDL and LDL mg/dL = mmol/L * 38.61; triglycerides mg/dL = mmol/L * 88.5; insulin µIU/mL = pmol/L * 0.1440; glucose mg/dL = mmol/L * 18. Conversion formulas to SI units from Quest Diagnostics.

**Table 2:** Prevalence of metabolic syndrome pre- and post-intervention.

Parameter	Enrolled	Pre	Post
	N = 14	N = 9	
Elevated blood pressure (SBP > 90th%-ile)	7 (50)	5 (56)	3 (33)
Obesity (WC > 90th%-ile)	14 (100)	9 (100)	8 (89)
Glucose intolerance (Fasting glucose ≥ 110 mg/dL [6.11 mmol/L])	0 (0)	0 (0)	0 (0)
Dyslipidemia (Triglycerides ≥ 110 mg/dL [1.24 mmol/L])	6 (43)	5 (56)	3 (33)
Dyslipidemia (HDL-C ≤ 40 mg/dL [1.04 mmol/L])	8 (57)	6 (66)	5 (56)
Subjects with metabolic syndrome * (≥3 risk factors)	7 (50)	6 (66)	2 (22)

Data are presented as number of subjects (%). * Based on definition of pediatric metabolic syndrome by Cook et al., 2003. No significant difference in prevalence pre- versus post-intervention (*p* = 0.07).

## Supplementary Materials

The following are available online at https://www.mdpi.com/2227-9067/6/9/99/s1, Table S1: Individual results, raw data.
