# Supplementary material for: Go Girls!—Dance-Based Fitness to Increase Enjoyment of Exercise in Girls at Risk for PCOS
Source: Children (Basel). 2019 Sep 6;6(9):99. doi: 10.3390/children6090099 (PMC6769571; doi:10.3390/children6090099)
Supplement: Supplementary file 1 [file children-06-00099-s001.pdf]

Value 1 = baseline for enrolled subjects
Value 2 = baseline for completing subjects
Value 3 = post-intervention for completing subjects
Delta values refer to change between post and pre values for those completing study (Value 3 - Value 2)
SHBG = sex hormone binding globulin
MetS = Metabolic Syndrome Severity Score
LDL = low density lipoprotein
HDL = high density lipoprotein
TG = triglycerides
Assays were resulted using U.S. units and values were converted to SI units.
Conversion formulas to SI units from Quest Diagnostics Laboratory
Race: b=Black; h=Hispanic; c=Caucasian

Table S1. Individual results, raw data.

Subject	Age 1	Age 2	Race 1	Race 2	Weight 1 (kg)	Weight 2 (kg)	Weight 3 (kg)	delta weight	Height	BMI 1 (kg/m2)
1	12.0		c		93.6				152.2	40.4
2	14.0		c		120.5				164.6	44.5
3	17.3	17.3	c	c	95.8	95.8	98.3	2.5	155.3	39.7
4	12.0	12.0	c	c	81.5	81.5	83.9	2.5	157.5	32.8
5	14.0		c		152.6				170.2	52.7
6	11.0		c		82.8				153.6	35.1
7	12.0		b		78.7				158.5	31.3
8	10.0		h		76.5				154.2	32.2
9	12.0	12.0	c	c	58.3	58.3	61.5	3.2	149.3	26.1
10	12.0	12.0	c	c	112.8	112.8	119.4	6.6	168.9	39.6
11	9.0	9.0	c	c	55.1	55.1	64.6	9.5	142.0	27.3
12	13.0	13.0	h	h	98.3	98.3	97.1	-1.2	157.2	39.8
13	14.0	14.0	c	c	90.5	90.5	95.0	4.5	159.1	35.7
14	16.8	16.8	c	c	108.0	108.0	105.9	-2.1	166.8	38.8
15	16.0	16.0	c	c	78.0	78.0	73.5	-4.5	160.3	30.4
16	14.8	14.8	b	b	64.9	64.9	56.5	-8.4	163.2	24.4
17	8.0	8.0	c	c	41.9	41.9	42.2	0.4	127.5	25.8
18	17.1		b		103.4				164.8	38.1
19	9.0	9.0	b	b	54.2	54.2	58.3	4.1	141.1	27.2
20	16.3		c		89.5				165.4	32.7
21	14.6		c		110.0				159.7	43.1
22	14.9		b		85.7				168.8	30.1
23	16.9		b		90.2				168.8	31.7
24	13.6		h		68.2				152.8	29.2
25	10.0	10.0	c	c	62.3	62.3	62.3	0.0	144.7	29.8
26	14.0	14.0	c	c	85.2	85.2	83.9	-1.3	158.0	34.1
27	9.0	9.0	b	b	42.0	42.0	43.6	1.6	139.8	21.5
28	15.0	15.0	b	b	124.8	124.8	123.8	-1.0	165.0	45.8
Average	13.1	12.6	8/28 black	4/16 black	85.9	78.3	79.4	1.0	156.8	34.3
Std Dev	2.7	3.0	3/28 Hisp	1/16 Hisp	25.6	25.5	25.5	4.3	10.4	7.2
N	28	16	28	16	28	16	16	16	28	28

BMI 2 (kg/m2)	BMI 3 (kg/m2)	BMI% 1	BMI% 2	BMI% 3	DeltaBMI %	BMIz1	BMIz2	BMIz3	DeltaBMIz	SBP1	SBP2	SBP3	sbpz1	sbpz2	sbpz3
		100.0				2.71				122			1.51		
		100.0				2.70				112			0.14		
39.7	40.7	98.9	98.9	98.9	0.0	2.29	2.29	2.29	0.00	118	118	108	1.11	1.11	0.25
32.8	33.8	98.9	98.9	99.0	0.0	2.30	2.30	2.32	0.02	128	128	161	1.94	1.94	5.08
		99.8				2.88				136			2.27		
		100.0				2.58				108			0.13		
		99.0				2.26				109			0.11		
		99.5				2.53				130			2.23		
26.1	27.6	96.0	96.0	97.0	1.0	1.76	1.76	1.87	0.11	105	105	99	-0.03	-0.03	-0.6
39.6	41.9	99.7	99.7	99.7	0.1	2.68	2.68	2.76	0.08	150	150	121	3.74	3.74	0.98
27.3	32.0	99.0	99.0	99.6	0.6	2.33	2.33	2.64	0.30	103	103	120	0.00	0.00	1.63
39.8	39.3	99.6	99.6	99.5	0.0	2.62	2.62	2.60	-0.02	136	136	110	2.71	2.71	0.23
35.7	37.5	99.1	99.1	99.3	0.2	2.35	2.35	2.44	0.09	143	143	140	3.26	3.26	2.98
38.8	38.0	99.0	99.0	99.0	0.0	2.28	2.28	2.24	-0.04	140	140	143	2.57	2.57	2.86
30.4	28.6	96.4	96.4	94.6	-1.7	1.80	1.80	1.61	-0.18	119	119	100	0.79	0.79	-1.02
22.3	21.2	87.0	87.0	67.0	-20.0	1.13	1.13	0.43	-0.70	102	102	97	-0.84	-0.84	-1.31
25.8	26.0	99.0	99.0	99.0	0.0	2.35	2.35	2.37	0.02	113	113	102	1.42	1.42	0.37
		99.0				2.20									
27.2	29.3	99.0	99.0	99.0	0.0	2.32	2.32	2.48	0.16	109			0.6		
		97.7				2.00				119			0.64		
		100.0				2.62				110			0.05		
		97.0				1.86				112			-0.06		
		97.0				1.84				124			0.99		
		97.0				1.90				115			0.79		
29.8	29.8	99.0	99.0	99.0	0.0	2.38	2.38	2.38	0.00	119	119	128	1.43	1.43	2.29
34.1	33.6	98.7	98.7	98.5	-0.1	2.21	2.21	2.18	-0.03	129	129	115	82	82	70
21.5	22.3	95.0	95.0	96.0	1.0	1.60	1.60	1.75	0.15	101	101	95	-0.12	-0.12	-0.69
45.8	45.4	100.0	100.0	100.0	0.0	2.67	2.67	2.66	-0.01	133	133	128	2.05	2.05	1.57
32.3	32.9	98.2	97.8	96.6	-1.2	2.26	2.19	2.19	0.00	120.2	122.6	117.8	4.13	6.80	5.64
7.1	7.1	2.6	3.2	8.0	5.1	0.40	0.42	0.57	0.22	13.4	15.9	19.4	15.61	20.84	17.89
16	16	28	16	16	16	28	16	16	16	27	15	15	27	15	15

sbp%1	sbp%2	sbp%3	Deltasbp%	MetSz1	MetSz2	MetSz3	MetS%1	MetS%2	MetS%3	Waist Circ 1 (cm)	Waist Circ 2 (cm)	Waist Circ 3 (cm)	Delta waist circ (cm)	Insulin 1 (uIU/mL)	Insulin 2 (uIU/mL)
93										104.1					
55										123.8					
87	87	61	-26	1.432	1.432	1.183	92.37	92.37	88.14	118.7	118.7	123.4	4.7	12.4	12.4
97	97	100	3	1.339	1.339	2.197	90.96	90.96	98.61	109.2	109.2	112.1	2.9	45.3	45.3
99				1.936			97.36			142.0				30.9	
55				1.137			87.21			117.0				26.1	
54										77.5					
99				1.951			97.44			94.4				33.8	
49	49	27	-22	0.774	0.774	0.585	78.08	78.08	72.09	102.9	102.9	99.4	-3.5	20.4	20.4
100	100	84	-16	2.123	2.123	1.628	98.31	98.31	94.81	116.2	116.2	120.0	3.8	45.2	45.2
50	50	95	45	1.275	1.275	1.858	89.88	89.88	96.84	93.2	93.2	103.5	10.3	22.6	22.6
100	100	59	-41	1.681	1.681	1.265	95.35	95.35	89.69	121.5	121.5	115.8	-5.7	36.2	36.2
100	100	100	0	1.545	1.545	1.971	93.87	93.87	97.56	116.5	116.5	114.8	-1.7	65.1	65.1
99	99	100	1							132.2	132.2	130.6	-1.6	34.4	34.4
79	79	15	-64	0.657	0.657	-0.039	74.46	74.46	48.46	96.8	96.8	89.8	-7.0	5.8	5.8
20	20	9	-11							84.8	84.8	77.0	-7.8		
92	92	64	-28							80.1	80.1	77.5	-2.6		
										112.4					
73										91.0	91.0	94.0	3.0		
74				0.746			77.23			100.9				9.2	
52				1.086			86.11			130.5				30.3	
47										87.8					
89										95.0					
79										101.5					
92	92	99	7							97.3	97.3	93.4	-3.9		
97	97	73	-24	1.551	1.551	0.995	93.94	93.94	84.01	104.0	104.0	98.0	-6.0	12.1	12.1
45	45	24	-21							84.0	84.0	80.0	-4.0		
98	98	94	-4							129.0	129.0	118.1	-10.9		
76.8	80.3	66.9	-13.4	1.374	1.375	1.294	89.47	89.69	85.58	105.9	104.8	103.0	-1.9	28.7	30.0
23.3	26.0	33.5	24.7	0.463	0.448	0.715	7.89	8.04	16.24	17.0	16.3	17.0	5.5	15.9	18.6
27	15	15	15	14	9	9	14	9	9	28	16	16	16	15	10

Insulin 3 (uIU/mL)	Insulin 1 (pmol/L)	Insulin 2 (pmol/L)	Insulin 3 (pmol/L)	Glucose 1 (mg/dL)	Glucose 2 (mg/dL)	Glucose 3 (mg/dL)	Glucose 1 (mmol/L)	Glucose 2 (mmol/L)	Glucose 3 (mmol/L)	HOMA 1	HOMA 2	HOMA 3	Total Testosterone 1 (ng/dL)	Total Testosterone 2 (ng/dL)	Total Testosterone 3 (ng/dL)
20.7	86.1	86.1	143.8	86	86	86	4.78	4.78	4.78	2.63	2.63	4.40	12.4	12.4	58.2
50.2	314.6	314.6	348.6	85	85	78	4.72	4.72	4.33	9.51	9.51	9.67	10.5	10.5	95.8
	214.6			99			5.50			7.55			28.8		
	181.3			102			5.67			6.57			10.4		
	234.7			88			4.89			7.34			22.3		
31.5	141.7	141.7	218.8	81	81	84	4.50	4.50	4.67	4.08	4.08	6.53	10.0	10.0	23.8
42.5	313.9	313.9	295.2	87	87	93	4.83	4.83	5.17	9.71	9.71	9.76	11.6	11.6	92.9
32.1	157.0	157.0	222.9	90	90	90	5.00	5.00	5.00	5.02	5.02	7.13	43.2	43.2	10.0
28.1	251.4	251.4	195.2	75	75	81	4.17	4.17	4.50	6.70	6.70	5.62	69.8	69.8	10.0
64.8	452.1	452.1	450.0	77	77	92	4.28	4.28	5.11	12.38	12.38	14.72	85.4	85.4	22.2
48.1	238.9	238.9	334.1	92			5.11			7.81			59.4	59.4	46.9
5.5	40.3	40.3	38.2	76	76	81	4.22	4.22	4.50	1.09	1.09	1.10	21.7	21.7	10.0
	63.9			78			4.33			1.77			22.9		
	210.4			91			5.06			6.81			25.0		
12.4	84.0	84.0	86.1	80	80	75	4.44	4.44	4.17	2.39	2.39	2.30	10.0	10.0	10.0
33.6	199.0	208.0	233.3	85.8	81.9	84.4	4.77	4.55	4.69	6.09	5.95	6.80	29.6	33.4	38.0
18.2	110.6	129.3	126.4	8.2	5.3	6.3	0.45	0.30	0.35	3.24	3.88	4.18	24.1	28.8	34.0
10	15	10	10	15	9	9	15	9	9	15	9	9	15	10	10

SHBG 1 (nmol/L)	SHBG 2 (nmol/L)	SHBG 3 (nmol/L)	Free Testosterone 1 (pg/mL)	Free Testosterone 2 (pg/mL)	Free Testosterone 3 (pg/mL)	Free Testosterone 1 (pmol/L)	Free Testosterone 2 (pmol/L)	Free Testosterone 3 (pmol/L)	DHEA-S 1 (ug/dL)	DHEA-S 2 (ug/dL)	DHEA-S 3 (ug/dL)	DHEA-S 1 (umol/L)	DHEA-S 2 (umol/L)	DHEA-S 3 (umol/L)	HDL 1 (mg/dL)
26.3	26.3	68.7	2.5	2.5	6.4	8.7	8.7	22.2	204	204	246	5.53	5.53	6.67	40
13.2	13.2	11.4	2.9	2.9	28.3	10.1	10.1	98.2	164	164	209	4.44	4.44	5.66	44
9.5			8.8			30.5			524			14.20			43
22.6			2.3			8.0			142			3.85			39
16.7			5.6			19.4			134			3.63			32
39.5	39.5	31.2	1.6	1.6	4.4	5.6	5.6	15.3	28	28	17	0.75	0.75	0.46	37
13.2	13.2	12.0	3.2	3.2	27.0	11.1	11.1	93.7	39	39	42	1.07	1.07	1.13	37
33.2	33.2	28.9	7.7	7.7	1.9	26.7	26.7	6.6	63	63	53	1.71	1.71	1.43	35
16.9	16.9	12.8	17.7	17.7	2.8	61.4	61.4	9.7	157	157	167	4.25	4.25	4.53	30
14.4	14.4	19.3	23.2	23.2	5.2	80.5	80.5	18.0	204	204	178	5.53	5.53	4.82	46
33.6	33.6	6.7	10.6	10.6	15.7	36.8	36.8	54.5	97	97	40	2.63	2.63	1.08	
38.7	38.7	36.3	3.5	3.5	1.7	12.1	12.1	5.9	42	42	51	1.13	1.13	1.37	46
80.1			2.2			7.6			153			4.15			55
18.9			6.0			20.8			233			6.31			41
22.3	22.3	21.1	2.2	2.2	2.3	7.6	7.6	8.0	27	27	31	0.72	0.72	0.83	40
26.6	25.1	24.8	6.7	7.5	9.6	23.1	26.1	33.2	147	102	103	3.99	2.78	2.80	40
17.6	10.6	18.2	6.3	7.5	10.4	21.9	26.0	36.0	125	73	86	3.38	1.98	2.34	6
15	10	10	15	10	10	15	10	10	15	10	10	15	10	10	14

HDL 2 (mg/dL)	HDL 3 (mg/dL)	HDL 1 (mmol/L)	HDL 2 (mmol/L)	HDL 3 (mmol/L)	LDL 1 (mg/dL)	LDL 2 (mg/dL)	LDL 3 (mg/dL)	LDL 1 (mmol/L)	LDL 2 (mmol/L)	LDL 3 (mmol/L)	LDL/HDL 1	LDL/HDL 2	LDL/HDL 3	TG 1 (mg/dL)	TG 2 (mg/dL)
40	47	1.04	1.04	1.22	157	157	150	4.07	4.07	3.89	3.9	3.9	3.2	183	183
44	41	1.14	1.14	1.06	85	85	71	2.20	2.20	1.84	1.9	1.9	1.7	77	77
		1.11			75			1.94			1.7			64	
		1.01			80			2.07			2.1			65	
		0.83			92			2.38			2.9			150	
37	35	0.96	0.96	0.91	70	70	75	1.81	1.81	1.94	1.9	1.9	2.1	144	144
37	36	0.96	0.96	0.93	76	76	78	1.97	1.97	2.02	2.1	2.1	2.2	55	55
35	32	0.91	0.91	0.83	104	104	86	2.69	2.69	2.23	3.0	3.0	2.7	243	243
30	28	0.78	0.78	0.73	98	98	113	2.54	2.54	2.93	3.3	3.3	4.0	110	110
46	39	1.19	1.19	1.01	90	90	81	2.33	2.33	2.10	2.0	2.0	2.1	56	56
46	50	1.19	1.19	1.30	90	90	87	2.33	2.33	2.25	2.0	2.0	1.7	57	57
		1.42			76			1.97			1.4			87	
		1.06			71			1.84			1.7			66	
40	44	1.04	1.04	1.14	72	72	79	1.86	1.86	2.05	1.8	1.8	1.8	145	145
39	39	1.05	1.02	1.01	88	94	91	2.29	2.42	2.36	2.3	2.4	2.4	107	119
5	7	0.17	0.14	0.19	22	26	25	0.58	0.68	0.65	0.7	0.8	0.8	58	66
9	9	14	9	9	14	9	9	14	9	9	14	9	9	14	9

TG 3 (mg/dL)	TG 1 (mmol/L)	TG 2 (mmol/L)	TG 3 (mmol/L)	HbA1c 1 (%)	HbA1c 2 (%)	HbA1c 3 (%)	Total PACES 1 (max 80)	Total PACES 2 (max 80)	Total PACES 3 (max 80)	Average PACES 1	Average PACES 2	Average PACES 3
							48			3.00		
277	2.07	2.07	3.13	5.4	5.4	5.6	74	74	80	4.63	4.63	5.00
79	0.87	0.87	0.89	5.3	5.3	5.1	24	24	22	1.50	1.50	1.38
	0.72			6.0			49			3.06		
	0.73			5.8			80			5.00		
							59			3.69		
	1.69			5.3								
92	1.63	1.63	1.04	5.7	5.7	5.2	74	74	65	4.63	4.63	4.06
87	0.62	0.62	0.98	5.8	5.8	5.9	54	54	62	3.38	3.38	3.88
205	2.75	2.75	2.32	5.3	5.3	5.1	70	70	69	4.38	4.38	4.31
89	1.24	1.24	1.01	5.0	5.0	5.0	68	68	69	4.25	4.25	4.31
109	0.63	0.63	1.23	5.0	5.0	5.1	19	19	43	1.19	1.19	2.69
							53	53	58	3.31	3.31	3.63
43	0.64	0.64	0.49	5.3	5.3	4.9	76	76	80	4.75	4.75	5.00
							63	63	70	3.94	3.94	4.38
							76	76	79	4.75	4.75	4.94
							73			4.56		
	0.98			5.7			79			4.94		
	0.75			5.6			78			4.88		
							61			3.81		
							65			4.06		
							65			4.06		
							77	77	80	4.81	4.81	5.00
117	1.64	1.64	1.32	5.6	5.6	5.7	80	80	80	5.00	5.00	5.00
							71			4.44		
							76			4.75		
122	1.21	1.34	1.38	5.5	5.4	5.3	64	62	66	4.03	3.88	4.12
73	0.65	0.74	0.82	0.3	0.3	0.4	16	20	17	1.01	1.25	1.07
9	14	9	9	14	9	9	25	13	13	25	13	13
